# MicroRNA-29b attenuates non-small cell lung cancer metastasis by targeting matrix metalloproteinase 2 and PTEN

**DOI:** 10.1186/s13046-015-0169-y

**Published:** 2015-06-11

**Authors:** Hongyan Wang, Xiaoying Guan, Yongsheng Tu, Shaoqiu Zheng, Jie Long, Shuhua Li, Cuiling Qi, Xiaobin Xie, Huiqiu Zhang, Yajie Zhang

**Affiliations:** Department of Pathology, School of Basic Medical Science, Guangzhou Medical University, 195# Dongfeng West Road, Guangzhou, Guangdong 510182 People’s Republic of China; Department of Physiology, School of Basic Medical Sciences, Guangzhou Medical University, 195# Dongfeng West Road, Guangzhou, Guangdong 510182 People’s Republic of China

**Keywords:** miR-29b, NSCLC, Metastasis, MMP2, PTEN

## Abstract

**Background:**

Our pilot study using miRNA PCR array found that miRNA-29b (miR-29b) is differentially expressed in primary cultured CD133-positive A549 cells compared with CD133-negative A549 cells.

**Methods:**

Ten human non-small cell lung cancer (NSCLC) cell lines and samples from thirty patients with NSCLC were analyzed for the expression of miR-29b by quantitative RT-PCR. Bioinformatics analysis combined with tumor metastasis PCR array showed the potential target genes for miR-29b. miR-29b lentivirus and inhibitors were transfected into NSCLC cells to investigate its role on regulating cell proliferation which was measured by CCK-8 assay *in vitro* and nude mice xenograft tumor assay *in vivo*. Cell motility ability was evaluated by transwell assay. The target genes of miR-29b were determined by luciferase assay, quantitative RT-PCR and western blot.

**Results:**

Bioinformatics analysis combined with tumor metastasis PCR array showed that matrix metalloproteinase 2 (MMP2) and PTEN could be important target genes of miR-29b. The expression of miR-29b was down regulated in NSCLC tissues compared to the normal tissues. Clinicopathological analysis demonstrated that miR-29b had significant negative correlation with lymphatic metastasis. The gain-of-function studies revealed that ectopic expression of miR-29b decreased cell proliferation, migration and invasion abilities of NSCLC cells. In contrasts, loss-of-function studies showed that inhibition of miR-29b promoted cell proliferation, migration and invasion of NSCLC cells *in vitro*. Nude mice xenograft tumor assay confirmed that miR-29b inhibited lung cancer growth *in vivo*. High-invasion (A549-H) and low-invasion (A549-L) NSCLC cell sublines from A549 cells were created by using the repeated transwell assay aimed to confirm the effect of miR-29b on migration and invasion of NSCLC. Furthermore, the dual-luciferase reporter assay demonstrated that miR-29b inhibited the expression of the luciferase gene containing the 3’-UTRs of MMP2 and PTEN mRNA. Western blotting and quantitative RT-PCR indicated that miR-29b down-regulated the expression of MMP2 at the protein and mRNA levels.

**Conclusion:**

Taken together, our results demonstrate that miR-29b serves as a tumor metastasis suppressor, which suppresses NSCLC cell metastasis by directly inhibiting MMP2 expression. The results show that miR-29b may be a novel therapeutic candidate target to slow NSCLC metastasis.

**Electronic supplementary material:**

The online version of this article (doi:10.1186/s13046-015-0169-y) contains supplementary material, which is available to authorized users.

## Background

Lung cancer is characterized by a low survival and high relapse rate after surgery [1, 2]. NSCLC, the most frequently occurring category of lung cancer, accounts for approximately 80 % of all cases [3]. Tumor invasion and metastasis are the main factors responsible for NSCLC treatment failure [4, 5].

Emerging evidence has revealed that microRNAs (miRNAs) play key roles in various biological processes, including metastasis, proliferation, apoptosis, stress resistance, tumorigenesis, and cell differentiation [6, 7]. Compared with normal tissues, different tumors have distinct miRNA expression characteristics. It has been determined that abnormal miRNA expression is a critical carcinogenesis signal. In tumors, miRNAs perform functions similar to those of oncogenes and tumor suppressors. miRNA expression patterns are more finely regulated than those of proteins [8]. Given these findings, miRNA may have great potential as a biomarker in early tumor diagnosis and treatment targets [9]. Many studies have attempted to identify the metastasis-related miRNAs in metastatic tumors using miRNA microarrays analysis. For example, several miRNAs have been identified to be involved in development of NSCLC metastasis, including let-7 [10], miR-200 [11], miR-125b [12] and miR-10b [13]. Although miRNAs have been the subject of extensive research in recent years, the molecular regulatory mechanisms of miRNAs and their effects on cancer are not well understood.

In the present study, to screen the metastasis-related miRNAs of NSCLC, CD133-positive and CD133-negative subpopulation from human lung adnocarcinoma A549 cells were isolated through immunomagnetic bead separation method. CD133 has been considered a specific stem cell marker and NSCLC prognosis marker [14, 15]. CD133-positive cells have greater potential for proliferation, metastasis, and chemo-radioresistance [16–18]. Through bioinformatics analysis and miRNA PCR array and tumor metastasis PCR array, PTEN, ETV4, COL4A2 and MMP2 were logically been speculated as miR-29b target genes. Our results showed that miR-29b inhibited growth and metastasis of NSCLC cells *in vitro* and *in vivo*. Additionally, dual-luciferase reporter assay and western blot results further elucidated that the miR-29b inhibited the expression of the luciferase gene containing the 3’-UTRs of MMP2 and PTEN mRNA. While miR-29b down-regulated the expression of MMP2 at the protein and mRNA levels. The results showed that miR-29b maybe a novel therapeutic candidate target or strategy for seeking to control NSCLC metastasis.

## Methods

### Tissue samples, cell culture and animals

Information about tissue specimens, NSCLC cell lines and animals is given in the Additional file 1.

### Microarray screening of differentially expressed genes between CD133-positive/negative NSCLC cells

Detailed information about isolation of CD133-positive/negative A549 cells and microarray screening of differentially expressed genes is provided in the Additional file 1.

### Bioinformatics analysis

Using “miRNA” as the index word for predicting target genes in the TargetScan (www.targetscan.org), PicTar (www.pictar.org), and miRanda (www.microrna.org) databases, target genes were identified from overlapping results from the three databases. Subsequently, we extracted the overlap of these results with that of the tumor metastasis PCR array.

### Quantitative RT-PCR

Quantitative RT-PCR was performed using kits for U6 and mature miR-29b (ABI, Foster City, CA, USA), according to the manufacturer’s instructions. SYBR green real-time RT-PCR was performed to detect MMP2 and PTEN. Detailed information is provided in the Additional file 1.

### Transfection studies

All miRNA duplexes (Additional file 2: Table S1) were purchased from Genepharma (Shanghai, P.R. China). H460 cells were transfected with inhibitor or inhibitor NC at a final concentration of 100 nmol/L using Lipofectamine RNAiMAX instructions (Invitrogen). A549 subline stably expressing miR-29b (A549-miR-29b) and its control line (A549-NC) were established as described in the Additional file 1.

### Western blotting

The cells were lysed with radioimmunoprecipitation assay buffer (Beyotime, Shanghai, China). The antibodies used for western blotting are described in the Additional file 1.

### Plasmid construction

We purchased psiCHECK-2 plasmids from Promega (Madison, WI, USA). Human genome DNA was used as the template for the MMP2 3’ untranslated region (UTR) and PTEN 3’ UTR PCR. *Xho*I and *Not*I restriction sites were introduced at the 5’ ends of both the forward and reverse primers (Additional file 2: Table S1). Following double digestion, the linear psiCHECK-2 fragment was connected with the 3’ UTRs using T4 DNA ligase; positive clones were selected for sequencing validation after transformation. The sequencing-validated, target recombinant plasmid was designated psiCHECK-2-Wt-MMP2/PTEN-3’ UTR, and was used as a template for constructing psiCHECK-2-Mut-MMP2/PTEN-3’ UTR using antisense PCR and a site-specific mutagenesis kit (Toyobo, Osaka, Japan).

### Luciferase reporter activity assay

Plasmid (0.5 μg) and 50 nmol/L miR-29b mimic/mimic NC were cotransfected using Lipofectamine LTX reagent (Invitrogen). Three replicates and three parallel lines were used each time. Following 48-h transfection, luciferase activity was measured using a GloMax 20/20 Luminometer (Promega) according to the dual luciferase reporting system instructions. Relative luciferase activity was compared using the ratio of *Renilla reniformis* and firefly luciferase activity (Rn/Ff).

### Cell proliferation assay

To measure the effect of miRNA and inhibitor on cellular proliferation rates, cells were incubated in 10 % CCK-8 (DOJINDO) diluted in normal culture media at 37 °C until visual color conversion appears. Proliferation rates were determined at 24, 48, 72, 96, 120 h post-transfection, and quantification was done on a microplate reader set according to the manufacturer’s protocol.

### *In vitro* assays of migration and invasion

The 24-well Boyden chamber with 8-μm pore size polycarbonate membrane (Corning, NY) was used to analyze the migration and invasion of tumor cells. Details are in the Additional file 1.

### *In vivo* studies

H460 subline stably knockdown miR-29b (H460-LV-miR-29b inhibitor) and its control line (H460-LV-CON), were established as described in Additional file 1. Analysis for tumorigenicity was performed as described in Additional file 1.

### Statistical analysis

All data were analyzed using SPSS 13.0 (SPSS Inc, Chicago, IL, USA); A paired *t* test was used to investigate the difference in the expression level of miR-29b between normal and cancerous tissues. A 2-sample *t* test was used to analyse the clinicopathologic characteristics of miR-29b expression in the tissues of patients with NSCLC. Quantitative RT-PCR, CCK-8 assay, migration and invasion assay, and luciferase reporter assay were tested using 1-way analysis of variance for factorial design. *P* value < 0.05 was considered statistically significant.

## Results

### Screening and identifying the metastasis-related miRNAs and target genes of NSCLC

**Fig. 1:**
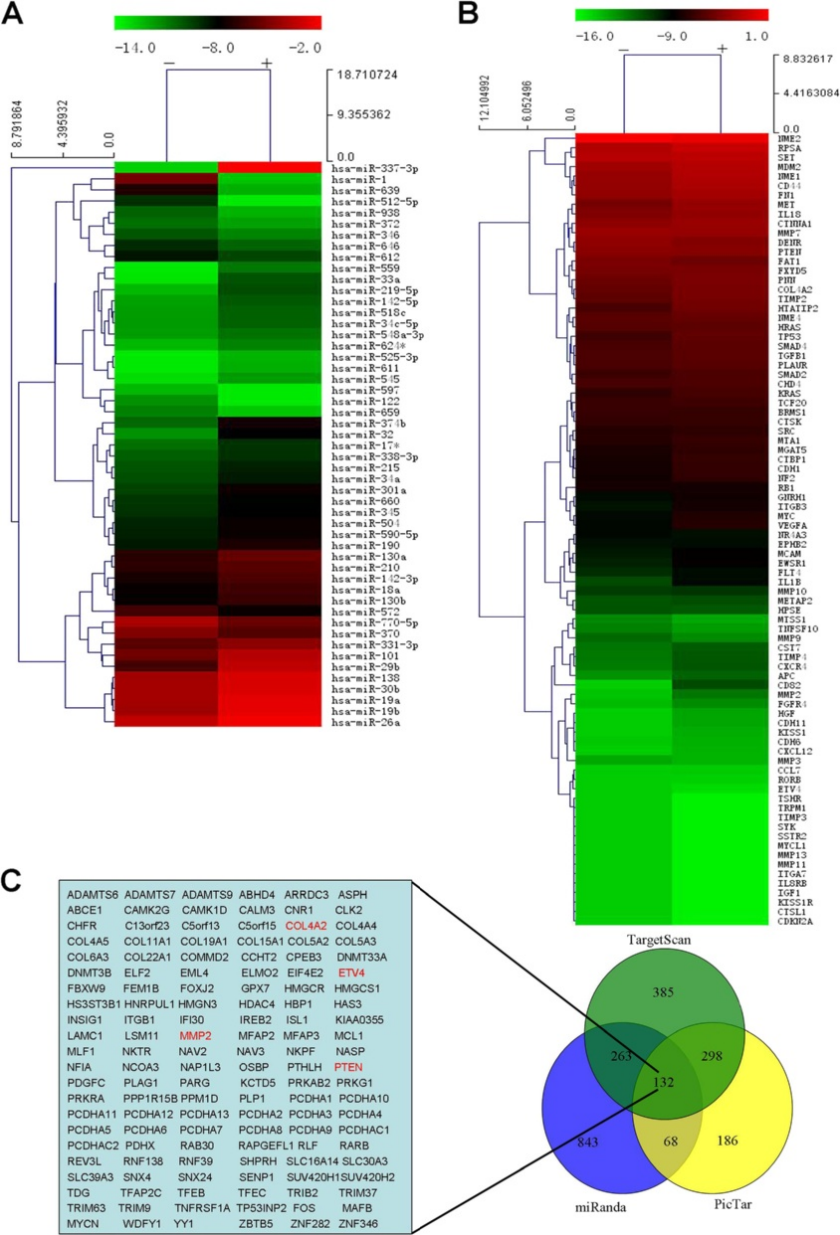
Integrated method for screening potential miRNAs and target genes related to NSCLC metastasis. Dendrogram of differentially expressed miRNAs (**a**) and metastasis-related genes (**b**) between primary cultured CD133-positive/negative lung adenocarcinoma cells. (**c)**, The overlap of target genes predicted by microRNA.org, TargetScan and Pictar datebases

To explore the miRNAs related to NSCLC metastasis, miRNA PCR array (MAH-3100A detected 376 human disease–related miRNA) were used to evaluate miRNA expression in primary cultured CD133-positive/negative A549 cells. Fourteen miRNAs were found up-regulated and thirty-seven miRNAs were down-regulated in CD133-positive cells (Fig. 1a, Additional file 3: Table S2). The human tumor metastasis PCR array (PAHS-028A detected 84 metastasis-related genes) was used to further determine the metastasis-related genes that could be controlled by CD133-regulated miRNAs. Nineteen metastasis-related genes were found up-regulated in CD133-positive cells (Fig. 1b, Additional file 4: Table S3). Finally, the target genes of significantly different miRNAs were predicted by bioinformatics analysis. The overlap genes were found between bioinformatics predicted analysis and tumor metastasis PCR array. Among the predicted target genes of the seven down-regulated miRNAs in CD133-positive A549 cells, the tumor metastasis PCR array contained four target genes of miR-29b (Fig. 1c). MiR-29b was down-regulated 7.6-fold in CD133-positive cells. however, its putative target genes *PTEN*, *ETV4*, *COL4A2*, and *MMP2* were up-regulated 1.11 to 4.2-fold. Based on PTEN and MMP2 were reported closely related to metastasis process, these two genes were further investigated to confirm their regulation by miR-29b in NSCLC.

### miR-29b is down-regulated in NSCLC tissues

**Fig. 2:**
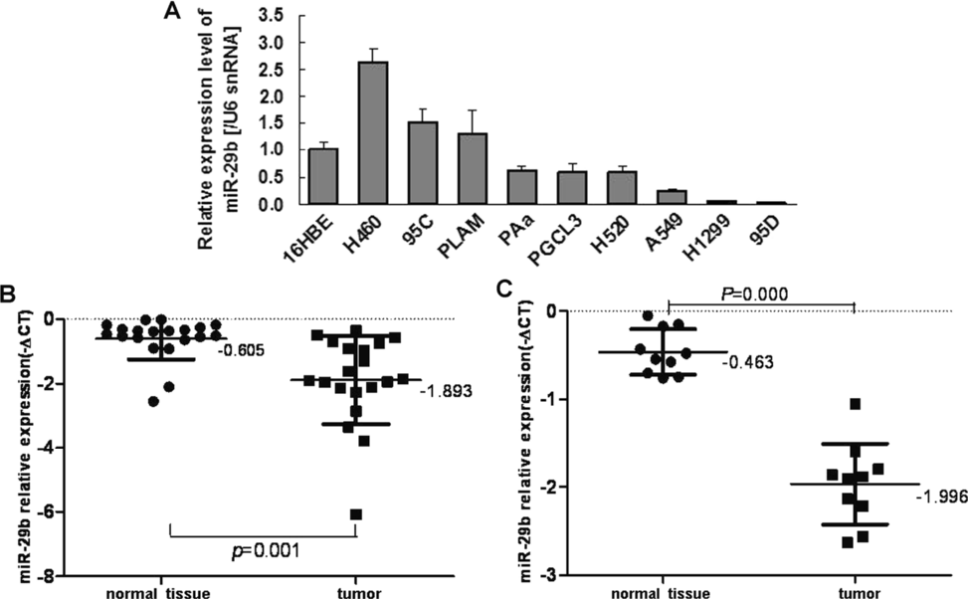
Expression of miR-29b in NSCLC cell lines and paired NSCLC tissues. **a**, Quantitative RT-PCR analysis of miR-29b expression levels in NSCLC cell lines and immortalized human bronchial epithelial cell line were shown relative to U6 snRNA as an internal control. **b**, Quantitative RT-PCR analysis of miR-29b levels in 20 pairs of paraffin-embedded NSCLC tissues. **c**, Quantitative RT-PCR analysis of miR-29b levels in 10 pairs of fresh NSCLC tissues

Quantitative RT-PCR results revealed that the expression levels of miR-29b were significantly higher in the H460 and 95C cell lines compared to 16HBE cell line, while the expression levels were lower in the PGCL3, PAa, H520, A549, H1299 and 95D cell lines (Fig. 2a). Twenty pairs of paraffin-embedded NSCLC tissues and normal tissues (Fig. 2b) and ten pairs of fresh NSCLC tissues and normal adjacent tissues (Fig. 2c) were also chosen to detect the expression levels of miR-29b, the results showed that the expression level of miR-29b in twenty cases of paraffin NSCLC tissues was (−1.893 ± 1.367), significantly lower than that in the adjacent lung tissue (−0.605 ± 0.639; *P* = 0.001, *t* = −3.817). The expression level of miR-29b in ten cases of fresh non-small cell lung cancer tissues was (−1.996 ± 0.460), significantly lower than that in the adjacent lung tissue (−0.463 ± 0.257; *P* < 0.001, *t* = −9.016).

Data present from Table 1 showed the clinicopathologic characteristics of miR-29b expression in NSCLC patients. There was no significant relationship of miR-29b expression with age (*P* = 0.578), gender (*P* = 0.862), histology (*P* = 0.625) and differentiation (*P* = 0.891); while miR-29b expression was found had significant relationships with lymphatic metastasis (*P* = 0.004) and clinic stage (*P* = 0.031). Spearman rank correlation analysis was applied to analyze the expression levels of miR-29b, tumor stage and lymphatic metastasis in NSCLC tissues. The expression of miR-29b was positively correlated with lymphatic metastasis (r = −0.547, *P* = 0.043). Based on our findings, the expression of miR-29b is down-regulated in NSCLC compared to normal tissues and significantly associated with metastasis.

**Table 1:** Clinicopathologic characteristics of miR-29b expression in NSCLC patients

Features	Case(*n*)	Percent	-△CT	*P*
Age				
>60	14	46.67	−2.044 ± 1.311	
= < 60	16	53.33	−1.807 ± 0.989	0.578
Gender				
Male	16	53.33	−1.883 ± 0.722	
Female	14	46.67	−1.957 ± 1.508	0.862
Histology				
Squamous cancer	4	13.33	−2.182 ± 0.858	
Adenocarcinoma	26	86.67	−1.877 ± 1.182	0.625
Differentiation				
Well + Moderate	21	70	−1.931 ± 1.344	
Poor	9	30	−1.886 ± 0.401	0.891
Clinic Stage				
I	10	33.33	−1.122 ± 0.638	
II	9	30	−1.881 ± 0.453	
III	11	36.67	−2.671 ± 1.398	0.004
Lymphatic Metastasis				
No	16	53.33	−1.505 ± 0.799	
Yes	14	46.67	−2.389 ± 1.302	0.031

### miR-29b suppresses cell proliferation, migration and invasion in A549 cells

**Fig. 3:**
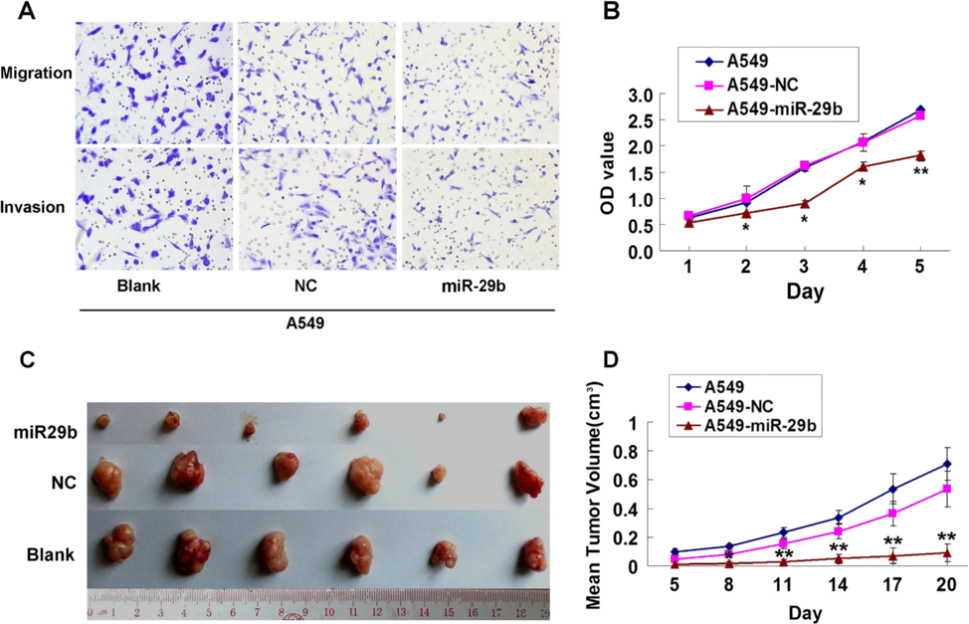
miR-29b inhibited cell proliferation, migration and invasion *in vitro*. **a**, In Matrigel invasion and transwell migration assay, LV-miR-29b infected A549 cells vs NC infected cells in a 200× light scope after crystal violet staining. Cells were counted in a light scope in four random views. **b**, miR-29b decreased cellular proliferation ability in A549 cells by CCK8 assay. *P < 0.05, **P < 0.01, compared with blank and NC groups. **c**, Photographs of subcutaneous tumors of mice injected with A549 cells that infected with LV-miR-29b compared to NC infected cells treatment. **d**, Subcutaneous tumors growth curves of each group after injection. ** *P* < 0.01, compared with blank and NC groups

Cell proliferation, migration, and invasion are key steps in tumor metastasis required by tumor cells for metastatic progression in target microenvironments. In A549 cells, after miR-29b lentivirus and negative control (NC) infection, a visible significant difference was seen with fewer miR-29b transfected cells counted than NC transfected cells in migration and invasion assays (Fig. 3a). Consistently, compared with negative controls, the proliferation abilities of A549-miR-29b cells were significantly decreased (Fig. 3b, *P < 0.05, **P < 0.01). Nude mice xenograft model was subsequently applied to evaluate the effect of miR-29b on tumorigenicity (Fig. 3c). Statistical analysis of the mean tumor volume (cm^3^) demonstated that miR-29b lentivirus infection inhibited the tumor growth comparing to the control groups (Fig. 3d, **P < 0.01). These findings might indicate that upregulation of miR-29b had a potential to inhibit of metastasis of NSCLC.

### miR-29b deficiency alters the metastasis ability of H460 cells

**Fig. 4:**
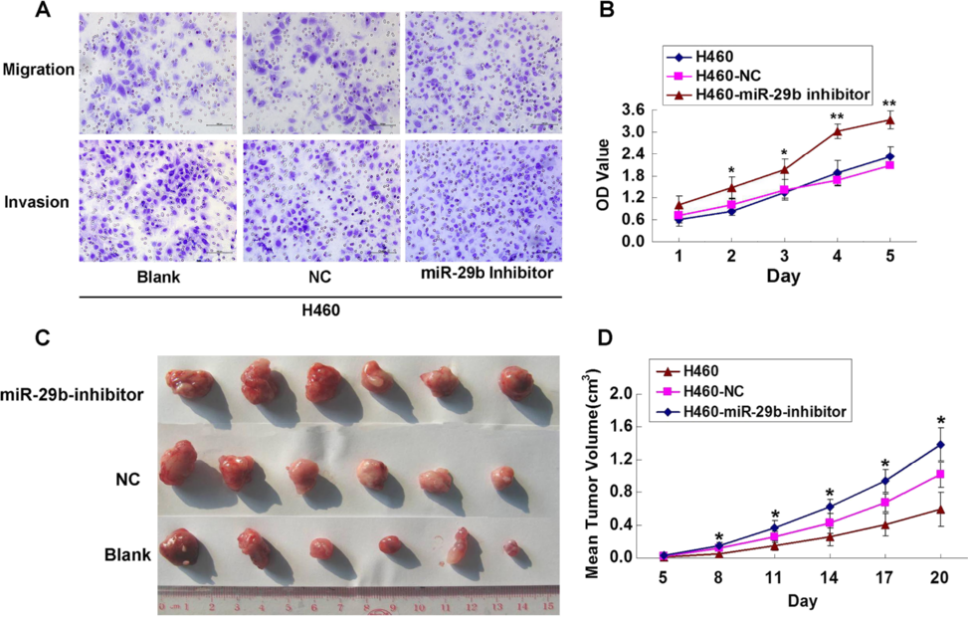
miR-29b deficiency altered the metastasis ability of H460 cells. **a**, In Matrigel invasion and transwell migration assay, LV-miR-29b inhibitor infected H460 cells vs NC infected cells in a 200× light scope after crystal violet staining. Cells were counted in a light scope in four random views. **b**, miR-29b inhibitor increased cellular proliferation ability in H460 cells by CCK8 assay. *P < 0.05, **P < 0.01, compared with blank and NC groups. **c**, Photographs of subcutaneous tumors of mice injected with H460 cells that infected with LV-miR-29b inhibitor compared to NC infected cells treatment. **d**, Subcutaneous tumors growth curves of each group after injection. **P < 0.01, compared with blank and NC groups

In the present study, we have observed an apparent endogenous expression of miR-29b in H460 cells. Therefore, miR-29b silencing with antisense oligonucleotides was administrated in H460 cells. Figure 4a showed that cell migration and invasion ability was promoted in miR-29b inhibitor group comparing to the control groups. miR-29b inhibitor also increased H460 cells proliferation in a time-dependent manner (Fig. 4b, *P < 0.05, **P < 0.01). Compared to H460 cells or H460-LV-NC cells group, tumor growth rates and tumor volumes of H460-LV-miR-29b-inhibitor cells group were significantly increased (Fig. 4c, d, *P < 0.05). Collectively, these observations suggested that miR-29b suppressed growth and metastasis of NSCLC cell *in vitro* and *in vivo*.

### Effect of miR-29b on cell migration and invasion ability in A549-L and A549-H cells

**Fig. 5:**
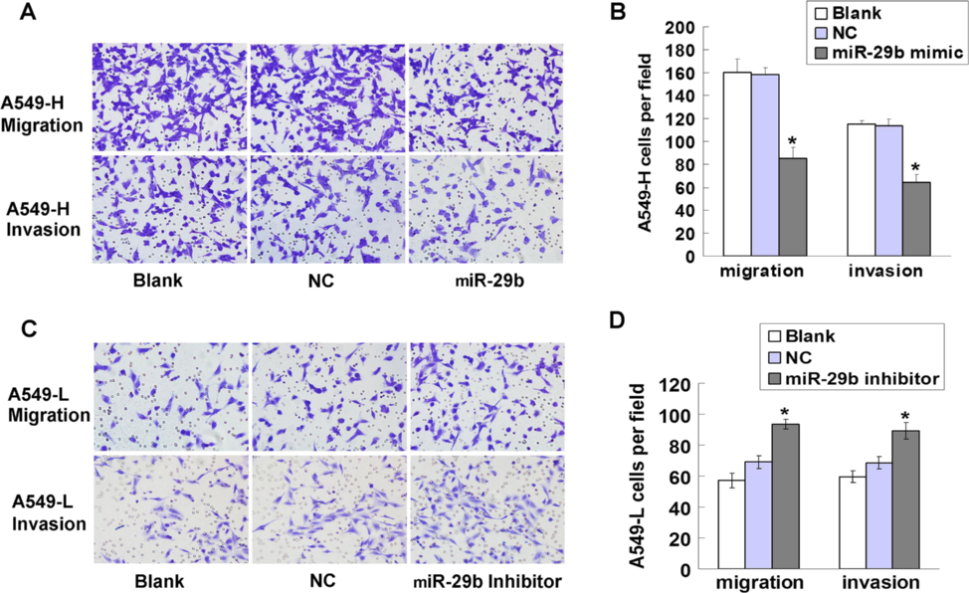
Effect of miR-29b on migration and invasion ability of A549-L and A549-H cells. **a**, In Matrigel invasion and migration assay, LV-miR-29b infected A549-H cells vs NC infected cells in a 200× light scope after crystal violet staining. **b**, Cells were counted in a light scope in four random views (*P < 0.05, *n* = 4). **c**, In Matrigel invasion and migration assay, miR-29b inhibitor infected A549-L cells vs NC infected in a 200× light scope after crystal violet staining. **d**, Cells were counted in a light scope in four random views (*P < 0.05, *n* = 4)

High-invasion (A549-H) and low-invasion (A549-L) NSCLC cell sublines from A549 cells were created by using the 10 times repeated transwell assay. Herein, the role of miR-29b in cell migration and invasion was evaluated these two kinds of cells. miR-29b was found down-regulated in A549-H cells and up-regulated in A549-L cells. The images showed that miR-29b overexpression inhibited A549-H cells migration and invasion (Fig. 5a). The result of statistical analysis was showed in Fig. 5b (*P < 0.05). As expected, miR-29b inhibitor promoted cell migration and invasion (Fig. 5c) of A549-L cells. The result of statistical analysis was showed in Fig. 5d (*P < 0.05). These data confirmed that miR-29b was a metastasis suppressor in NSCLC cells.

### MMP2 as a target gene of miR-29b in NSCLC

**Fig. 6:**
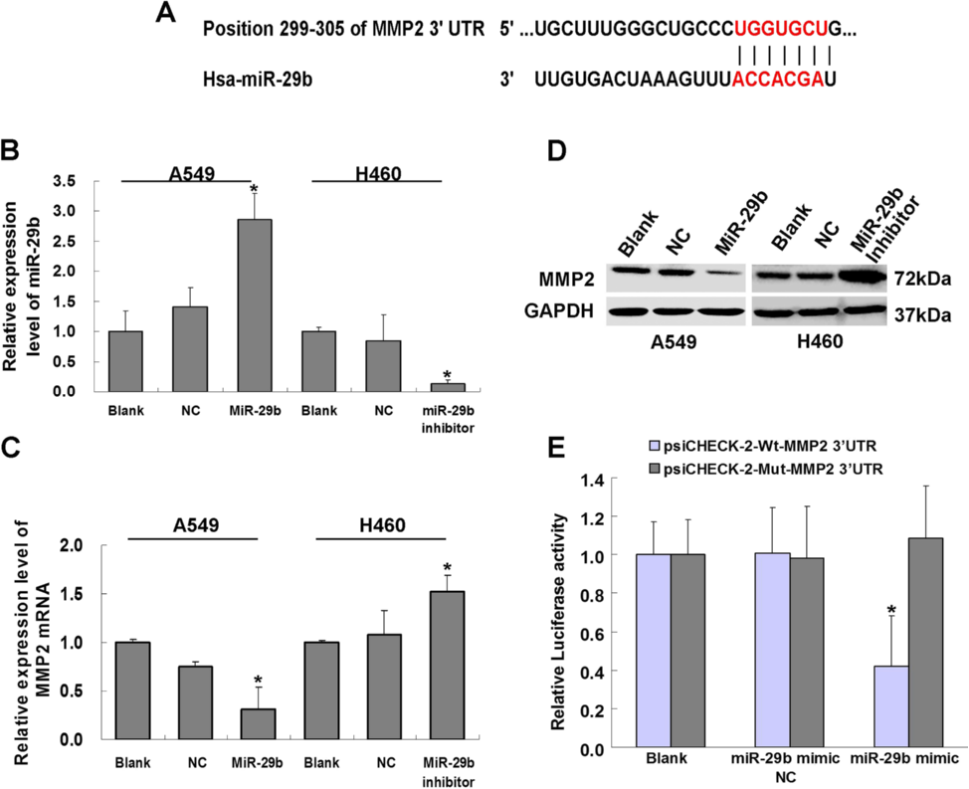
miR-29b directly targets 3’-UTR of the MMP2 gene. **a**, Predicted binding sites in the 3’-UTR of MMP2 mRNA and seed sequence of miR-29b by TargetScan. **b**, Quantitative RT-PCR analysis of miR-29b expression levels in A549 and H460 cell lines after infection LV-miR-29b or miR-29b inhibitor, respectively. **c**, Quantitative RT-PCR analysis of MMP2 mRNA expression levels in A549 and H460 cell lines after infection LV-miR-29b or miR-29b inhibitor, respectively. **d**, Western blots analysis of MMP2 expression levels in A549 and H460 cell lines following LV-miR-29b or miR-29b inhibitor infection, respectively. **e**, Dual luciferase activity indicating relative luciferase activity following co-transfection with psiCHECK-2-Wt-MMP2-3’UTR or psiCHECK-2-Mut-MMP2-3’UTR and miR-29b mimic. Results are presented as means ± SEM (*P < 0.05, *n* = 3)

To further explore the mechanisms of miR-29b which suppresses lung cancer cell invasion and metastasis, we analyzed probable down stream tumor metastasis-related genes. Computational prediction was used to find out the most likely target genes. The taregets of miR-29b were analyzed by the TargetScan, PicTar and MiRanda databases. The analysis revealed that miR-29b bound to the MMP2 3’ UTR with a partially complementary pattern (Fig. 6a). After A549 cells were infected with lentivirus LV-miR-29b, the expression of miR-29b was increased significantly compared with the NC and Blank groups (Fig. 6b, *P < 0.05). As expected, the expression of MMP2 mRNA was down-regulated significantly (Fig. 6c, *P < 0.05). Moreover, western blot results showed that MMP2 protein expression was decreased significantly (Fig. 6d). Similar results were obtained when miR-29b inhibitor was transfected into the H460 cells, the miR-29b expression level decreased and MMP2 mRNA was up-regulated significantly after 48 h transfection compared with the NC group and Blank groups (Fig. 6b, c, *P < 0.05). MMP2 protein expression was increased after 72 h transfection (Fig. 6d). These findings indicated that miR-29b regulated MMP2 expression negatively. To verify whether MMP2 is a direct target of miR-29b, the 3’UTR of MMP2 cDNA was cloned into the downstream region of the luciferase reporter gene (psiCHECK-2-Wt-MMP2-3’UTR) and co-transfected this vector into 293-T cells with miR-29b mimic (Additional file 5: Figure S1A, B). The luciferase reporter gene study further confirmed that miR-29b bound directly to wild-type MMP2 3’ UTR to inhibit the luciferase activity. To confirm the sequence-specific repression of miR-29b, we designed mutated versions of psiCHECK-2-Wt-MMP2-3’UTR carrying 4-bp substitutions in miR-29b target site (psiCHECK-2-Mut-MMP2-3’UTR) (Additional file 5: Figure S1C, D). There was no inhibition effect on the following site-specific mutagenesis of the miR-29b MMP2 3’ UTR binding sites (Fig. 6e, *P < 0.05), indicating that miR-29b directly regulated the target gene MMP2 negatively in NSCLC.

### miR-29b affected PTEN expression by binding directly with the PTEN 3’ UTR

**Fig. 7:**
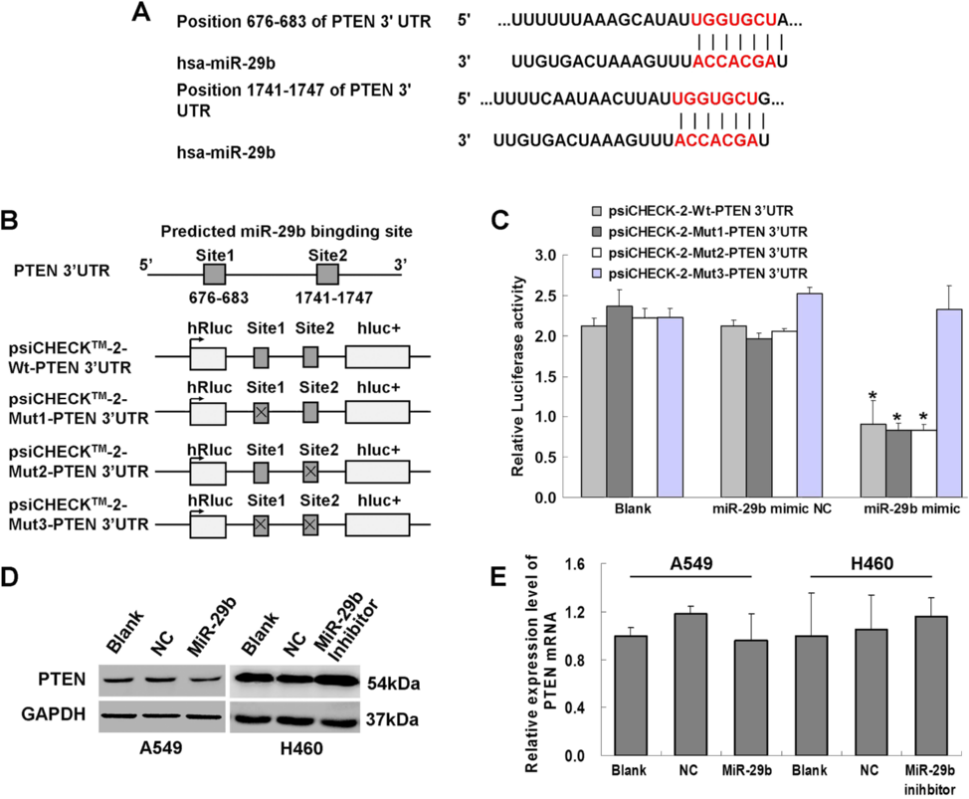
miR-29b directly targets 3’-UTR of the PTEN gene. **a**, Predicted binding sites in the 3’-UTR of PTEN mRNA and seed sequence of miR-29b by TargetScan. **b**, Schematic diagrams of miR-29b and PTEN 3’ UTR binding and wild-type and mutated psiCHECK-2-PTEN-3’UTR sequences. **c**, Relative luciferase activity evaluated by dual luciferase reporter genes following co-transfection with psiCHECK-2-Wt-PTEN-3’UTR or psiCHECK-2-Mut1-3-PTEN-3’UTR and miR-29b mimic, respectively. Results are presented as means ± SEM (*P < 0.05, *n* = 3). **d**, Western blots analysis of PTEN expression levels in A549 and H460 cell lines following LV-miR-29b or miR-29b inhibitor infection, respectively. **e**, Quantitative RT-PCR analysis of PTEN mRNA expression levels in A549 and H460 cell lines following LV-miR-29b or miR-29b inhibitor infection, respectively

TargetScan indicated that miR-29b had two highly conserved PTEN 3’ UTR binding sites (Fig. 7a). Luciferase reporter activity assay was used to determine whether miR-29b regulates PTEN directly via the software-predicted binding sites. Figure 7b depicted the insertion sites of wild-type and mutant PTEN 3’ UTR into the constructed plasmids. The 1500-bp fragment of the 3’UTR region of PTEN mRNA that included the predicted miR-29b recognition site was subcloned and inserted into a luciferase reporter plasmid (Additional file 6: Figure S2A, B). Two miR-29b binding sites in the 3’UTR region of PTEN were mutated to obtain psiCHECK-2-Mut1-3-PTEN-3’UTR plasmid (Additional file 6: Figure S2C–F). Following detection, the luciferase activity in the co-transfected with wild-type PTEN-luc reporter and miR-29b mimic group was significantly decreased compared with that in the blank or NC groups (*P < 0.05). The luciferase activity in the co-transfected with mutant-type1 or mutant-type2 PTEN-luc reporter and miR-29b mimic group was also significantly decreased compared with that in the blank or NC groups (*P < 0.05). However, there were no significant changes in the co-transfected with mutant-type3 PTEN-luc reporter and miR-29b mimic group and the blank or NC groups (Fig. 7c, *P < 0.05). This result proved that miR-29b bound directly to both PTEN 3’UTR binding sites. Therefore, PTEN was a direct target of miR-29b. The influence of miR-29b on PTEN mRNA and protein expression levels were also evaluated in the A549 and H460 cells, There were no obvious changes on PTEN mRNA and protein expression levels in LV-miR-29b infected A549 cells or in miR-29b inhibitor transfected H460 cells compared with the NC or Blank groups (Fig. 7d, e).

## Discussion

In the present study, we provided evidence that miR-29b expression in high-metastatic CD133-positive A549 lines was down-regulated when compared to miR-29b expression in paired low-metastatic CD133-negtive A549 cell lines, miR-29b was confirmed directly targeted 3’-UTR of PTEN and MMP2 mRNAs and down-regulated MMP2 protein expression to suppress lung cancer metastasis *in vitro* and *in vivo*.

As a MMP superfamily member, MMP2 specifically degrades type IV collagen, a major component of the extracellular matrix and basal lamina, and is a major factor in tumor invasion and angiogenesis [19]. It has been demonstrated that high MMP2 expression is an independent prognostic factor in NSCLC and is closely related to clinical stage, pathological grade, lymphatic metastasis, and prognosis [20]. The regulatory mechanisms of a miRNA could differ among different microenvironments, miR-29b is upregulated in metastatic breast cancer tissues and indolent lymphocytic leukemia, functioning as an oncogene [21, 22]. However, miR-29b is down-regulated in lung carcinoma tissues [23]. In our study, low-level expression of miR-29b in NSCLC tissues was significantly associated with lymphatic metastasis. We performed gain-of-function in A459 cells and loss-of-function in H460 cells of miR-29b. Our data demonstrated that miR-29b inhibited *in vitro* cell proliferation, invasion and migration and *in vivo* suppressed NSCLC growth in a nude mice xenograft model. Furthermore, the dual-luciferase reporter assay demonstrated that miR-29b inhibited the expression of luciferase gene containing the 3’-UTR of PTEN and MMP2. Western blotting indicated that miR-29b down-regulated the endogenous protein expression of MMP2. Based on these results, It’s concluded that miR-29b was related to metastasis in NSCLC.

PTEN regulates tumor cell growth, cell cycle, apoptosis, and metastasis by regulating multiple signal transduction pathways negatively [24, 25]. All three databases used (TargetScan, PicTar, miRanda) identified the two PTEN 3’ UTR miR-29b binding sites. Both sites were conserved among different species and fully complementary to the miR-29b seed sequence, corresponding to the basic rules for predicting miRNA target genes [26]. Our present results showed that miR-29b bound directly to the two PTEN 3’ UTR binding sites and PTEN was a miR-29b target gene. Through miR-29b overexpression or knockdown analysis, the fact was determined that miR-29b variations were not accompanied with the alteration of PTEN expression. As multiple miRNAs could regulate the same target gene [27], we speculate that other miRNAs could also bind directly to the PTEN 3’ UTR and regulating PTEN expression. Several research reported that PTEN function as a target gene of miR-21 [28], miR-214 [29], miR-494 [30], miR-26a [31], miR-144 [32] and miR-153 [33]. of these, miR-21, miR-214, and miR-494 are upregulated in NSCLC. Another reason that might explain our contrasting findings was that miR-29b directly inhibits CDC42 and p85α to activate p53 expression [34]. P53 activates PTEN transcription, binding directly to the PTEN promoter and activating PTEN expression [35]. PTEN gene was not only indirectly regulated by miR-29b-p53-PTEN positively, but also directly regulated by miR-29b negatively. The inhibition of Sp1 by miR-29b resulted in the upregulation of PTEN in tongue squamous cell carcinoma [36].

## Conclusion

In summary, our studies demonstrated that down-regulated miR-29b expression was found to be associated with increased MMP2 expression in CD133-positive NSCLC cells through microarrays and bioinformatics analysis. miR-29b played a strong inhibitory role in tumor metastasis. We provided important evidence that miR-29b could suppress NSCLC cells proliferation, migration and invasion by targeting the 3’-UTR of MMP2 and PTEN mRNA to down-regulate MMP2 protein expression. Our findings provided novel evidence for the involvement of miR-29b in NSCLC metastasis, and suggested that miR-29b could be a potential new target for treatment of NSCLC metastasis.

## Consent

The patient consent of Written informed consent was obtained from the patient for the publication of this report and any accompanying images.

## Additional files

Additional file 1: **Supplementary Materials and Methods.**

Additional file 2: Table S1. Sequences of RNA and DNA Oligonucleotides.

Additional file 3: Table S2. Fifty-one miRNAs differentially expressed in CD133+ A549 cells versus CD133- A549 cells.

Additional file 4: Table S3. Changes in relative expression for tumor metastasis genes between CD133+ and CD133- A549 cells.

Additional file 5: Figure S1. Construction of mutant 3’UTR-MMP2-luc vector.

Additional file 6: Figure S2. Construction of mutant 3’UTR-PTEN-luc vector.

## References

[1] Subramaniam S, Thakur RK, Yadav VK, Nanda R, Chowdhury S, Agrawal A (2013). Lung cancer biomarkers: state of the art. J Carcinog.

[2] Hayano T, Garg M, Yin D, Sudo M, Kawamata N, Shi S (2013). SOX7 is down-regulated in lung cancer. J Exp Clin Cancer Res.

[3] MacDonagh L, Gray SG, Finn SP, Cuffe S, O’Byrne KJ, Barr MP (2014). The emerging role of microRNAs in resistance to lung cancer treatments. Cancer Treat Rev.

[4] Steeg PS (2003). Metastasis suppressors alter the signal transduction of cancer cells. Nat Rev Cancer.

[5] Huang J, Song H, Liu B, Yu B, Wang R, Chen L (2013). Expression of Notch-1 and its clinical significance in different histological subtypes of human lung adenocarcinoma. J Exp Clin Cancer Res.

[6] Tie J, Pan Y, Zhao L, Wu K, Liu J, Sun S (2010). MiR-218 inhibits invasion and metastasis of gastric cancer by targeting the Robo1 receptor. PLoS Genet.

[7] Zhu C, Zhao Y, Zhang Z, Ni Y, Li X, Yong H (2014). MicroRNA-33a inhibits lung cancer cell proliferation and invasion by regulating the expression of beta-catenin. Mol Med Rep.

[8] Boutros PC, Lau SK, Pintilie M, Liu N, Shepherd FA, Der SD (2009). Prognostic gene signatures for non-small-cell lung cancer. Proc Natl Acad Sci U S A.

[9] Ma MZ, Kong X, Weng MZ, Cheng K, Gong W, Quan ZW (2013). Candidate microRNA biomarkers of pancreatic ductal adenocarcinoma: meta-analysis, experimental validation and clinical significance. J Exp Clin Cancer Res.

[10] Yun J, Frankenberger CA, Kuo WL, Boelens MC, Eves EM, Cheng N (2011). Signalling pathway for RKIP and Let-7 regulates and predicts metastatic breast cancer. EMBO J.

[11] Roy SS, Gonugunta VK, Bandyopadhyay A, Rao MK, Goodall GJ, Sun LZ (2014). Significance of PELP1/HDAC2/miR-200 regulatory network in EMT and metastasis of breast cancer. Oncogene.

[12] Li Y, Chao Y, Fang Y, Wang J, Wang M, Zhang H (2013). MTA1 promotes the invasion and migration of non-small cell lung cancer cells by downregulating miR-125b. J Exp Clin Cancer Res.

[13] Ma L (2010). Role of miR-10b in breast cancer metastasis. Breast Cancer Res.

[14] Mizugaki H, Sakakibara-Konishi J, Kikuchi J, Moriya J, Hatanaka KC, Kikuchi E (2014). CD133 expression: a potential prognostic marker for non-small cell lung cancers. Int J Clin Oncol.

[15] Nakamura M, Zhang X, Mizumoto Y, Maida Y, Bono Y, Takakura M (2014). Molecular characterization of CD133+ cancer stem-like cells in endometrial cancer. Int J Oncol.

[16] Zhang H, Yang N, Sun B, Jiang Y, Hou C, Ji C (2014). CD133 positive cells isolated from A549 cell line exhibited high liver metastatic potential. Neoplasma.

[17] Bertolini G, Roz L, Perego P, Tortoreto M, Fontanella E, Gatti L (2009). Highly tumorigenic lung cancer CD133+ cells display stem-like features and are spared by cisplatin treatment. Proc Natl Acad Sci U S A.

[18] Hou Y, Zou Q, Ge R, Shen F, Wang Y (2012). The critical role of CD133(+)CD44(+/high) tumor cells in hematogenous metastasis of liver cancers. Cell Res.

[19] Halbersztadt A, Halon A, Pajak J, Robaczynski J, Rabczynski J, St Gabrys M (2006). The role of matrix metalloproteinases in tumor invasion and metastasis. Ginekol Pol.

[20] Qian Q, Wang Q, Zhan P, Peng L, Wei SZ, Shi Y (2010). The role of matrix metalloproteinase 2 on the survival of patients with non-small cell lung cancer: a systematic review with meta-analysis. Cancer Invest.

[21] Gebeshuber CA, Zatloukal K, Martinez J (2009). miR-29a suppresses tristetraprolin, which is a regulator of epithelial polarity and metastasis. EMBO Rep.

[22] Pekarsky Y, Croce CM (2010). Is miR-29 an oncogene or tumor suppressor in CLL?. Oncotarget.

[23] Fabbri M, Garzon R, Cimmino A, Liu Z, Zanesi N, Callegari E (2007). MicroRNA-29 family reverts aberrant methylation in lung cancer by targeting DNA methyltransferases 3A and 3B. Proc Natl Acad Sci U S A.

[24] Song MS, Salmena L, Pandolfi PP (2012). The functions and regulation of the PTEN tumour suppressor. Nat Rev Mol Cell Biol.

[25] de Assis LV, Isoldi MC (2014). The function, mechanisms, and role of the genes PTEN and TP53 and the effects of asbestos in the development of malignant mesothelioma: a review focused on the genes’ molecular mechanisms. Tumour Biol.

[26] Lewis BP, Shih IH, Jones-Rhoades MW, Bartel DP, Burge CB (2003). Prediction of mammalian microRNA targets. Cell.

[27] Chen Y, Gao D, Huang L (2015). *In vivo* delivery of miRNAs for cancer therapy: challenges and strategies. Adv Drug Deliv Rev.

[28] Zhang JG, Wang JJ, Zhao F, Liu Q, Jiang K, Yang GH (2010). MicroRNA-21 (miR-21) represses tumor suppressor PTEN and promotes growth and invasion in non-small cell lung cancer (NSCLC). Clin Chim Acta.

[29] Wang YS, Wang YH, Xia HP, Zhou SW, Schmid-Bindert G, Zhou CC (2012). MicroRNA-214 regulates the acquired resistance to gefitinib via the PTEN/AKT pathway in EGFR-mutant cell lines. APJCP.

[30] Liu Y, Lai L, Chen Q, Song Y, Xu S, Ma F (2012). MicroRNA-494 is required for the accumulation and functions of tumor-expanded myeloid-derived suppressor cells via targeting of PTEN. J Immunol.

[31] Liu B, Wu X, Wang C, Liu Y, Zhou Q, Xu K (2012). MiR-26a enhances metastasis potential of lung cancer cells via AKT pathway by targeting PTEN. Biochim Biophys Acta.

[32] Zhang LY, Ho-Fun Lee V, Wong AM, Kwong DL, Zhu YH, Dong SS (2013). MicroRNA-144 promotes cell proliferation, migration and invasion in nasopharyngeal carcinoma through repression of PTEN. Carcinogenesis.

[33] Wu Z, He B, He J, Mao X (2013). Upregulation of miR-153 promotes cell proliferation via downregulation of the PTEN tumor suppressor gene in human prostate cancer. Prostate.

[34] Park SY, Lee JH, Ha M, Nam JW, Kim VN (2009). miR-29 miRNAs activate p53 by targeting p85 alpha and CDC42. Nat Struct Mol Biol.

[35] Poon JS, Eves R, Mak AS (2010). Both lipid- and protein-phosphatase activities of PTEN contribute to the p53-PTEN anti-invasion pathway. Cell Cycle.

[36] Jia LF, Huang YP, Zheng YF, Lyu MY, Wei SB, Meng Z (2014). miR-29b suppresses proliferation, migration, and invasion of tongue squamous cell carcinoma through PTEN-AKT signaling pathway by targeting Sp1. Oral Oncol.

