# Supplementary material for: MicroRNA-29b attenuates non-small cell lung cancer metastasis by targeting matrix metalloproteinase 2 and PTEN
Source: J Exp Clin Cancer Res. 2015 Jun 11;34(1):59. doi: 10.1186/s13046-015-0169-y (PMC4469413; doi:10.1186/s13046-015-0169-y)
Supplement: Supplementary file 1 — Supplementary Materials and Methods. [file 13046_2015_169_MOESM1_ESM.doc]

**Additional file 1:**

**Supplementary Materials and Methods**

**Tissue specimens, cell lines and animals**

10 fresh and 20 formalin-fixed paraffin-embedded specimens of NSCLC tissues and corresponding matched normal tissues were collected from 30 patients who underwent resection for NSCLC at the Department of General Surgery in Guangzhou University Hospital (Guangzhou, China) in 2010; none of the patients received prior radiotherapy or chemotherapy. The fresh samples were collected immediately after resection, snap-frozen in liquid nitrogen, and stored at -80°C until needed. Informed consent was obtained from each patient and the study was approved by the Institute Research Ethics committee. We purchased the following cell lines from the Shanghai Cell Bank of the Chinese Academy of Science (Shanghai, China): A549, PAa, PGCL3, PLAM, H1299, H460, H520, 95D, 95C (human NSCLC); 16HBE (human immortalized bronchial epithelial cells); and 293T (human kidney cells). Cells were subcultured serially in RPMI 1640 complete medium supplemented with 10% fetal bovine serum at 37°C in a culture chamber containing 5% CO_2_. Log-phase cells were used in the experiments. Female BALB/c nude mice aged 5–6 weeks were purchased from the Guangdong Laboratory Animal Centre (Guangzhou, China). All procedures were performed according to the internationally accepted ethical guidelines.

**Microarray screening of differentially expressed genes between CD133-positive/negative NSCLC cells**

The magnetic bead isolation kit and magnetic isolation system were from Miltenyi Biotec (Bergisch Gladbach, Germany). Log-phase A549 cells were prepared as single-cell suspensions; approximately 10^7^ cells were suspended in 300 μL buffer, and 100 μL anti-Fc receptor blocker and 100 μL CD133 magnetic beads were added. Samples were incubated in darkness at 4°C for 30 min after sufficient resuspension. Samples were washed in buffer, resuspended and transferred to the positive separation (MS) column, which was placed in the separator. The column was washed with two volumes of buffer to elute CD133-negative cells after cell suspensions had been drained. Buffer (500 μL) was added, and the eluent containing CD133-positive cells was collected after the separation core had been matched to the separation column and the core quickly removed. The negative separation (LD) column was then placed in the separator. The eluent collected from the positive sorting process was transferred to the negative separation column and washed with buffer. The collected eluent contained CD133-negative cells.

CD133-positive and -negative cell suspensions sorted by the MS and LD columns, respectively, were centrifuged to sediment the cells; 1 mL TRIzol (Invitrogen, Carlsbad, CA, USA) was added to 5–10 × 10^6^ cells. The extracted RNA was purified using an RNeasy MinElute Cleanup Kit (Qiagen, Hilden, Germany). The Purity of the RNA is assessed by spectrophotometry (A260/A280 > 1.8) and the integrity was evaluated with denaturing agarose gel electrophoresis. Complementary DNA (cDNA) synthesis was carried out according to reverse transcription (RT)-PCR Array First Strand Kit (SABiosciences, Frederick, MD, USA) instructions. The cDNA was used in RT-PCR amplification after being added to the Human Tumor Metastasis PCR Array (PAHS-028A, SABiosciences) functional gene microarray RT profiler and miRNA microarray Human Genome RT² miRNA PCR Array (PAHS-3100A, SABiosciences). Data were analyzed using the comparative threshold (ΔΔCt) method. Differentially expressed genes (DEGs) had 2^-ΔΔCt^ ≥ 2.0 or ≤0.5.

**Quantitative Real-Time Polymerase Chain Reaction(Quantitative RT -PCR)**

Total RNA was extracted with a mirVana miRNA Isolation Kit (ABI, Foster City, CA, USA). The TaqMan stem-loop RT-PCR method was used to assess the expression of miRNAs with kits from Applied Biosystems (Foster City, CA,USA). The primers for miR-29b were obtained from Applied Biosystems (Foster City, California, USA). SYBR green real-time RT-PCR was performed to detect MMP2 and PTEN. The primers for MMP2 and PTEN were obtained from TaKaRa (Dalian, China). Data are presented as fold differences relative to either GAPHD for MMP2 and PTEN or U6 for miRNA based on calculations of 2^-ΔΔCt^. All primer sequences in this study are listed in Supplementary Table S1.

**Establishment of miR-29b stably expressing and knockdown cell line**

The recombinant lentivirus LV-mir-29b encoding mir-29b-1 and LV-NC(control), the recombinant lentivirus LV-miR-29b-inhibitor silencing the miR-29b and LV-CON(control) were purchased from Genecheme (Shanghai, P.R. China). All lentiviral particles contains the EGFP gene. Lentiviral particles were used to infect A549 cells, Colonies with GFP expression were selected by limiting dilution assay of 96-well plate to expand culture. Finally, A549 subline with miR-29b overexpression (A549-miR-29b) and negative control line (A549-NC) were respectively established for further investigation. H460 subline stably knockdown miR-29b (H460-LV-miR-29b-inhibitor) and its control line (H460-NC) were established as follow: H460 were infected with LV-miR-29b-inhibitor or LV-CON at a MOI of 10, the infection efﬁciency was about 90% as assessed by microscopy of GFP ﬂuorescence.

**Western blotting**

Following 72-h transfection, cells were dissociated with precooled radioimmunoprecipitation lysis solution on ice to extract proteins. Protein concentration was measured with a bicinchoninic acid assay. We performed 10% sodium dodecyl sulfate-polyacrylamide gel electrophoresis using 20 μg protein, and then samples were transferred to a nitrocellulose membrane and blocked at room temperature for 2 h. Rabbit polyclonal antibodies (matrix metalloproteinase 2 [MMP2], 1:1,000; glyceraldehyde-3-phosphate dehydrogenase [GAPDH], 1:1,000) (Gene Tech) were added to the membrane and incubated at 4°C overnight. The secondary antibody (1:1,000, Gene Tech) was added and incubated at 37°C for 1 h after washing three times with Tris-buffered saline (TBS). Membranes were washed three times with TBS prior to colorimetric detection using electrochemiluminescence. Radiographic figures were detected using a Vilber Lourmat electrophoresis transilluminator (Bio-Rad, Hercules, CA, USA); areas and gray values were measured using Quantity One (Bio-Rad).

**Isolation of highly invasive and weakly invasive cells using Transwell chambers**

A549 cells were isolated in Transwell chambers according to their differential invasiveness. In all, 2×10^5^ cells were plated in the top chamber where the membrane was coated with 30 μg of Matrigel (24-well insert; 8-mm pore size; Corning Costar Corp). The cells were plated in serum-free medium, whereas medium that was supplemented with serum was used as a chemoattractant in the lower chamber. Following an incubation for 24 h at 37°C, the cells that had migrated through the membranes and attached to the lower-chamber compartments were harvested aseptically and expanded for second-round selection. After a ten-round selection, the subline of cells that failed to invade the membranes in all selection rounds was designated A549-L, and the subline that was able to migrate through the membranes was designated A549-H.

**In Vitro Assays of Migration and Invasion**

The migration and invasion assays were done in 24-well transwell chamber with 8 μm pore size polycarbonate membrane (Corning, NY). For invasion assay, the membrane was coated with 30 μg Matrigel (RD Biosciences) to form a matrix barrier. Twenty-four hours after transfection with RNA duplex, tumor cells (2×10^5^ A549 or H460 in 200 μl serum-free medium) were added to the upper compartment of the chamber, while the lower compartment was filled with 700 μl of DMEM containing 10% FBS. After incubation at 37°C for 24h, tumor cells remaining on the upper surface of the membrane were removed. The cells on the lower surface of the membrane were fixed, stained with crystal violet and then counted under a light microscope. For migration assay, only one fifth of cells were applied to the transwell chamber without Matrigel.

**In vivo studies**

Animal xenograft model studies were performed according to institutional guidelines. A549, A549-miR-29b and A549-NC viable cells (2×10^6^), H460, H460-LV-miR-29b-inhibitor, H460-NC viable cells (1×10^6^) were suspended in 200 μl PBS and then injected subcutaneously into the left flank or right flank of 5-week-old female BALB/c nude mice (six mice per group), respectively. Tumor diameters were measured after 5-6 days from injection and then every 3 days. At 25 days after injection, mice were killed and tumors were weighted after necropsy. Tumor volume was calculated as follows: length × width^2^× 1/2.
