# Supplementary material for: MicroRNA-29b attenuates non-small cell lung cancer metastasis by targeting matrix metalloproteinase 2 and PTEN
Source: J Exp Clin Cancer Res. 2015 Jun 11;34(1):59. doi: 10.1186/s13046-015-0169-y (PMC4469413; doi:10.1186/s13046-015-0169-y)
Supplement: Supplementary file 2 — Sequences of RNA and DNA Oligonucleotides. [file 13046_2015_169_MOESM2_ESM.doc]

**Additional file 2:**

**Table S1** Sequences of RNA and DNA Oligonucleotides

| **Name** | **Sense Strand (5' - 3')** | **Antisense Strand (5' - 3')** |
| --- | --- | --- |
| **MiRNA Duplexes** | | |
| miR-29b mimic | UAGCACCAUUUGAAAUCAGUGUU | CACUGAUUUCAAAUGGUGUUAUU |
| miR-29b mimic NC | UUCUCCGAACGUGUCACGUTT | ACGUGACACGUUCGGAGAATT |
| miR-29b Inhibitors | AACACUGAUUUCAAAUGGUGCUA |  |
| miR-29b Inhibitors NC | CAGUACUUUUGUGUAGUACAA |  |
| **Primers for RT-PCR** |  |  |
| GAPHD | GCACCGTCAAGGCTGAGAAC | TGGTGAAGACGCCAGTGGA |
| MMP2 | CTCATCGCAGATGCCTGGAA | TTCAGGTAATAGGCACCCTTGAAGA |
| PTEN | TAGAGCGTGCAGATAATGACAAGGA | TGAACTGCTAGCCTCTGGATTTGA |
| **Primers for 3'UTR Cloning** | | |
| MMP2 Wt 3'UTR | AGTCTCGAGTCCACTGCCTTCGATACAC | AGTGCGGCCGCTTCAACTAATAATGGCCTTTT |
| MMP2 Mut 3'UTR | TAATATTGCCACACTTCAGGCTCTTCTCCTTT | GGGCAGCCCAAAGCAGGGCTGCGTTGAA |
| PTEN Wt 3'UTR | TCTCGAGCAACTGAAGTGGCTAAAGAG | AGTGCGGCCGCTGAAGTTCTGCCTAATCTA |
| PTEN Mut1 3'UTR | CATTTTTTTTTAAAGCATATACCACGAAGAAA  AGGCAGCTAAAGGAA | TTCCTTTAGCTGCCTTTTCTTCGTGGTATATGC  TTTAAAAAAAAATG |
| PTEN Mut2 3'UTR | TTCCATTTTCAATAACTTATACCACGAGAAAT  TGTTCACTAGCTGTG | CACAGCTAGTGAACAATTTCTCGTGGTATAA  GTTATTGAAAATGGAA |

Wt, Wild-type; Mut, mutation
