# Supplementary material for: MicroRNA-29b attenuates non-small cell lung cancer metastasis by targeting matrix metalloproteinase 2 and PTEN
Source: J Exp Clin Cancer Res. 2015 Jun 11;34(1):59. doi: 10.1186/s13046-015-0169-y (PMC4469413; doi:10.1186/s13046-015-0169-y)
Supplement: Supplementary file 3 — Fifty-one miRNAs differentially expressed in CD133+ A549 cells versus CD133- A549 cells. [file 13046_2015_169_MOESM3_ESM.doc]

**Additional file 3:**

**Table S2 Fifty-one miRNAs differentially expressed in CD133-positive A549 cells versus CD133-negative A549 cells**

| **miRNA ID** | **Chromosomal Localization** | **Fold Change** | **Type** |
| --- | --- | --- | --- |
| hsa-miR-346 | 10q23.2 | 2.12 | up |
| hsa-miR-612 | 11q13.1 | 2.26 | up |
| hsa-miR-210 | 11p15.5 | -2.01 | down |
| hsa-miR-659 | 22q13.1 | 3.14 | up |
| hsa-miR-597 | 8p23.1 | 2.16 | up |
| hsa-miR-130a | 11q12.1 | -2.62 | down |
| hsa-miR-512-5p | 19q13.42 | 20.50 | up |
| hsa-miR-938 | 10p11.23 | 3.80 | up |
| hsa-miR-30b | 8q24.2 | -2.40 | down |
| hsa-miR-370 | 14q32.2 | 2.16 | up |
| hsa-miR-372 | 19q13.42 | 2.30 | up |
| hsa-miR-190 | 15q22.2 | -2.38 | down |
| hsa-miR-33a | 22q13.2 | -10.91 | down |
| hsa-miR-504 | Xq26.3 | -2.18 | down |
| hsa-miR-215 | 1q41 | -2.38 | down |
| hsa-miR-770-5p | 14q32.2 | 2.83 | up |
| hsa-miR-32 | 9q31.3 | -10.62 | down |
| hsa-miR-26a | 3p22.2 | -2.26 | down |
| hsa-miR-572 | 4p15.33 | 2.55 | up |
| hsa-miR-646 | 20q13.33 | 2.57 | up |
| hsa-miR-611 | 11q12.2 | -2.55 | down |
| hsa-miR-18a | 13q31.3 | -2.60 | down |
| hsa-miR-660 | Xp11.23 | -2.27 | down |
| hsa-miR-559 | 2p21 | -6.39 | down |
| hsa-miR-525-3p | 3p26.1 | -2.35 | down |
| hsa-miR-19b | 13p31.3 | -3.07 | down |
| hsa-miR-142-3p | 17q22 | -2.36 | down |
| hsa-miR-639 | 19p13.12 | 31.45 | up |
| hsa-miR-624* | 14q12 | -2.38 | down |
| hsa-miR-122 | 18q21.31 | 4.08 | up |
| hsa-miR-34a | 1p36.23 | -2.34 | down |
| hsa-miR-142-5p | 17q22 | -3.31 | down |
| hsa-miR-19a | 13q31.3 | -2.84 | down |
| hsa-miR-130b | 22 | -2.26 | down |
| hsa-miR-34c-5p | 11q23.1 | -2.49 | down |
| hsa-miR-1 | 20q13.33 | 174.04 | up |
| hsa-miR-338-3p | 17q25.3 | -2.15 | down |
| hsa-miR-138 | 3q21.32 | -2.47 | down |
| hsa-miR-331-3p | 12q22 | -2.29 | down |
| hsa-miR-590-5p | 7q11.23 | -2.01 | down |
| hsa-miR-219-5p | 6p21.23 | -5.91 | down |
| hsa-miR-518c | 19q13.42 | -2.65 | down |
| hsa-miR-337-3p | 14q32.2 | -2024.95 | down |
| hsa-miR-101 | 1p31.3 | -3.02 | down |
| hsa-miR-345 | 14q32.2 | -2.21 | down |
| hsa-miR-374b | Xq13.2 | -7.97 | down |
| hsa-miR-545 | X | -2.87 | down |
| hsa-miR-29b | 7q32.3 | -7.24 | down |
| hsa-miR-548a-3p | 6 | -2.02 | down |
| hsa-miR-301a | 17q22 | -3.80 | down |
| hsa-miR-17* | 13q31.3 | -2.80 | down |
