# Supplementary material for: MicroRNA-29b attenuates non-small cell lung cancer metastasis by targeting matrix metalloproteinase 2 and PTEN
Source: J Exp Clin Cancer Res. 2015 Jun 11;34(1):59. doi: 10.1186/s13046-015-0169-y (PMC4469413; doi:10.1186/s13046-015-0169-y)
Supplement: Supplementary file 4 — Changes in relative expression for tumor metastasis genes between CD133+ and CD133- A549 cells. [file 13046_2015_169_MOESM4_ESM.doc]

**Additional file 4:**

**Table S3 Changes in relative expression for tumor metastasis genes between CD133+ and CD133- A549 cells.**

| **RefSeq** | **Gene Symbol** | **Gene Description** | **Chromosomal Localization** | **Fold Change** |
| --- | --- | --- | --- | --- |
| NM_002231  NM_004530  NM_000576  NM_002467  NM_003376  NM_006410  NM_000546  NM_000212  NM_002011  NM_000038  NM_000245  NM_001328  NM_004360 NM_000268 NM_005243 NM_000601 NM_001797 NM_003467  NM_000269 | CD82  MMP2  IL1B  MYC  VEGFA  HTATIP2  TP53  ITGB3  FGFR4  APC  MET  CTBP1  CDH1  NF2  EWSR1  HGF  CDH11  CXCR4  NME1 | CD82 molecule  Matrix metallopeptidase 2  Interleukin 1, beta  V-myc myelocytomatosis viral oncogene homolog  Vascular endothelial growth factor A  HIV-1 Tat interactive protein 230kDa  Tumor protein p53  Integrin, beta 3  Fibroblast growth factor receptor 4  Adenomatous polyposis coli  Met proto-oncogene  C-terminal binding protein 1  Cadherin 1, E-cadherin (epithelial)  Neurofibromin 2 (merlin)  Ewing sarcoma breakpoint region 1  Hepatocyte growth factor  Cadherin 11,OB-cadherin (osteoblast)  Chemokine (C-X-C motif) receptor 4  Non-metastatic cells 1, protein (NM23A) expressed in | 11p11.2  16q13-q21  2q14  8q24.21  6p12  11p15.1  17p13.1  17q21.32  5q35.1-qter  5q21-q22  7q31  7q31  4p16  16q22.1  22q12.2  7q21.1  16q21  2q21  17q21.3 | 12.0  4.2  3.4  3.1  3.1  2.8  2.6  2.5  2.5  2.5  2.4  2.3  2.3  2.3  2.2  2.2  2.1  2.1  2.1 |
