# Supplementary material for: MicroRNA-29b attenuates non-small cell lung cancer metastasis by targeting matrix metalloproteinase 2 and PTEN
Source: J Exp Clin Cancer Res. 2015 Jun 11;34(1):59. doi: 10.1186/s13046-015-0169-y (PMC4469413; doi:10.1186/s13046-015-0169-y)
Supplement: Supplementary file 5 — Construction of mutant 3’UTR-MMP2-luc vector. [file 13046_2015_169_MOESM5_ESM.doc]

**Additional file 5: Figure S1**

**
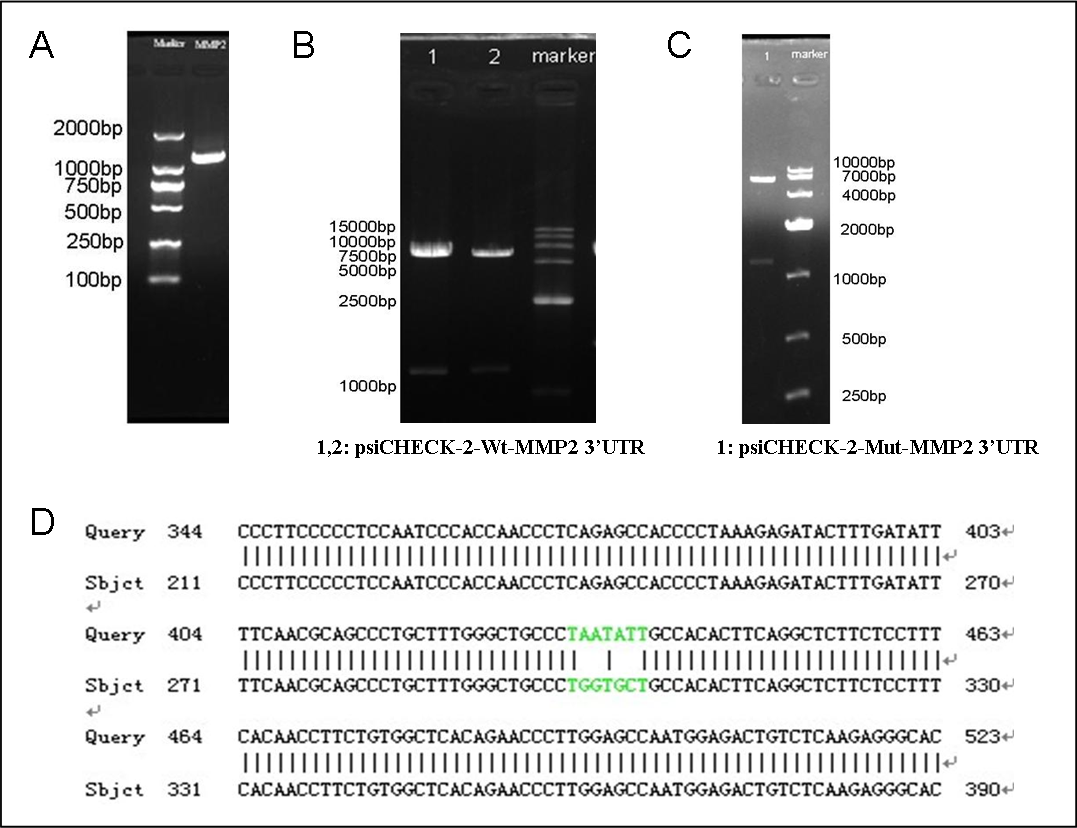
**

**Figure S1**. Construction of mutant 3’UTR-MMP2-luc vector. (A) The MMP2 3’UTR fragment was ampliﬁed by PCR. (B) Identification of psiCHECK-2-Wt-MMP2 3’UTR by restriction analysis. (C) Identification of psiCHECK-2-Mut-MMP2 3’UTR by restriction analysis. (D) The sequence of mutated binding site miR-29b in the 3’UTR region of MMP2.
