# Supplementary material for: MicroRNA-29b attenuates non-small cell lung cancer metastasis by targeting matrix metalloproteinase 2 and PTEN
Source: J Exp Clin Cancer Res. 2015 Jun 11;34(1):59. doi: 10.1186/s13046-015-0169-y (PMC4469413; doi:10.1186/s13046-015-0169-y)
Supplement: Supplementary file 6 — Construction of mutant 3’UTR-PTEN-luc vector. [file 13046_2015_169_MOESM6_ESM.doc]

**Additional file 6: Figure S2**


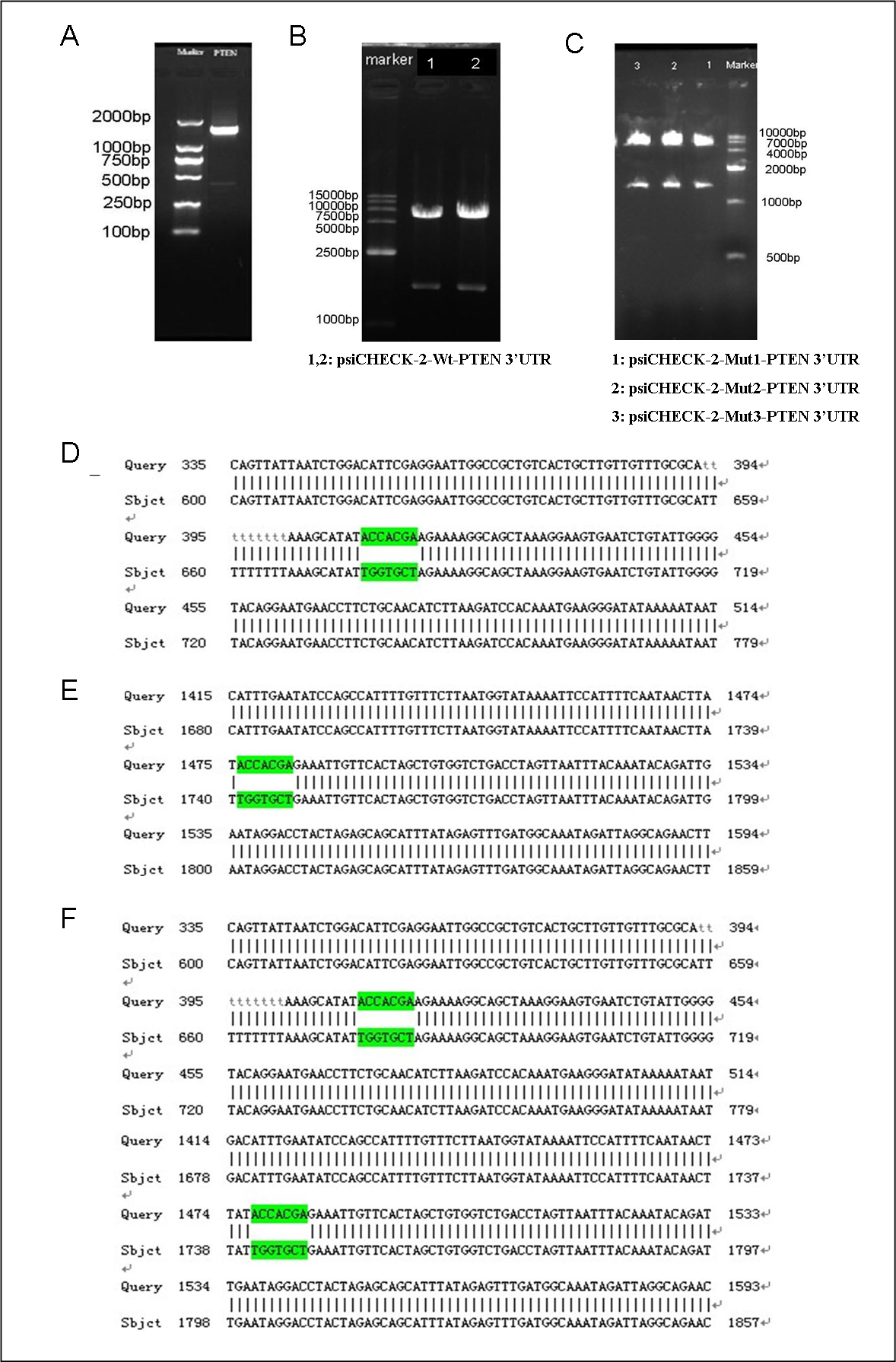


**Figure S2**. Construction of mutant 3’UTR-PTEN-luc vector. (A) The PTEN 3’UTR fragment was ampliﬁed by PCR. (B) Identification of psiCHECK-2-Wt-PTEN 3’UTR by restriction analysis. (C) Identification of psiCHECK-2-Mut-MMP2 3’UTR by restriction analysis. (D) Partial sequencing map containing the binding site of psiCHECK-2- Mut-1-PTEN 3’UTR, psiCHECK-2- Mut-2-PTEN 3’UTR(E), psiCHECK-2- Mut-3-PTEN 3’UTR(F) with miR-29b
